# Rationale and study protocol for We-PAP: a randomized pilot/feasibility trial of a couples-based intervention to promote PAP adherence and sleep health compared to an educational control

**DOI:** 10.1186/s40814-022-01089-x

**Published:** 2022-08-06

**Authors:** Kelly Glazer Baron, Allyson Gilles, Krishna M. Sundar, Brian R. W. Baucom, Kevin Duff, Wendy Troxel

**Affiliations:** 1 Division of Public Health, Department of Family and Preventive Medicine, University of Utah, 375 Chipeta Way, Salt Lake City, UT 84013 USA; 2 Division of Pulmonary & Critical Care Medicine, Department of Medicine, University of Utah, 26 N, 1900 E, Salt Lake City, UT 84132 USA; 3 Department of Psychology, University of Utah, 380 S. 1530 E., Room 502, Salt Lake City, UT 84112 USA; 4 Department of Neurology, University of Utah, 650 Komas Drive, Salt Lake City, UT 106A84108 USA; 5 RAND Corporation, 4570 Fifth Ave #600, Pittsburgh, PA 15213 USA

**Keywords:** Obstructive sleep apnea (OSA), Couple, Positive airway pressure (PAP), Adherence, Sleep, Transdiagnostic, BBTI, Cognitive behavioral, CBTi, Treatment, Alzheimer’s disease

## Abstract

**Background:**

Obstructive sleep apnea (OSA) is a serious health condition that affects approximately 30-50% of older adults and contributes to risk for cardiometabolic disorders and dementia. Despite the well-documented role of partners in treatment seeking and adherence to positive airway pressure (PAP), treatments for OSA have nearly exclusively focused on the patient and current treatments for OSA do not address co-existing sleep problems such as insomnia that are prevalent in both patients with OSA and their partners. Therefore, the goal of this study is to develop and test a novel couples-based sleep health intervention to promote adherence to PAP and improve sleep health of the couple.

**Methods:**

We are conducting a two-arm, parallel group, single blind, randomized controlled pilot/feasibility trial to compare our novel couples-based sleep health intervention (We-PAP) to an information control group (IC). We-PAP is based on a transdiagnostic model and uses a dyadic approach including increasing effective partner support, communication skills, and couple-level goal-setting. We-PAP involves 3 sessions and delivered via telehealth in weekly sessions. The IC includes standardized patient educational materials. Both groups receive the usual follow-up with their medical team. The study involves assessments at pre-treatment, post-intervention (approximately 1 month after starting PAP and completing We-PAP sessions or IC) and 3 months after starting PAP. Our main outcomes are feasibility and acceptability ratings. Secondary outcomes include comparing We-PAP to IC for PAP adherence, sleep quality (self-report and objective) and cognitive measures.

**Discussion:**

We-PAP is the first couples-based transdiagnostic sleep health intervention for patients with OSA and their partners. Results of this study will be used to inform the design of a subsequent fully powered clinical trial. If successful, this intervention could significantly advance current clinical practice in the treatment of OSA and sleep health more comprehensively in older adults. Moreover, this intervention may be useful for improving sleep in other aging populations with multiple sleep and other health problems, including patients with chronic illnesses or those at risk for Alzheimer’s disease and their caregivers.

**Trial registration:**

NCT04759157. Date of registration: February 8, 2021. URL of trial registry record.

## Background

Obstructive sleep apnea (OSA) is a major public health problem that affects 30% to 50% of older adults^1^ and is associated with significant morbidity and mortality. Older adults with OSA have a significantly increased risk for cardiovascular disease [2] and stroke [3, 4] and have five times the risk for Alzheimer’s disease (AD) and related dementias [5, 6]. The physiological consequences of OSA, including intermittent hypoxia and oxidative stress are key mechanisms that drive neurodegeneration and AD pathophysiology [7, 8]. Given that 61% of adults share a bed with a partner [9], the consequences of OSA, including fragmented sleep, reduced quality of life, and increased marital conflict, affect both the patient and partner [10, 11]. Further, given that sleep fragmentation is also mechanistically linked with increased risk for cognitive decline and is a symptom experienced by both the patient with OSA and their bedpartner [12], the public health consequences of OSA are far greater than just that experienced by the patient alone.

The first-line treatment for OSA, positive airway pressure (PAP), is highly effective at treating OSA symptoms in the patient and improving both patient and bedpartner sleep. There is a dose-response relationship between PAP adherence and improvements in both patient and bedpartner sleep [13], daytime sleepiness, and QOL, as well as reductions in OSA patients’ cardiometabolic and AD risk factors, including hypertension [14, 15]. PAP treatment may slow cognitive decline in patients with dementia [16]. Unfortunately, however, up to 80% of patients are non-adherent [15, 17, 18]. Patterns of PAP use in the first 30 days strongly predict later adherence [19, 20] and reimbursement of this treatment hinges on documenting adherence at 30–90 days. Therefore, interventions focusing on early use among new PAP users are vital to treatment success and to promote patient and bedpartner health.

There is a strong scientific premise for targeting PAP adherence at the couple-level. First, a consistent body of evidence demonstrates that couples’ sleep is highly interdependent, meaning that sleep in one partner affects and is affected by the other partner’s sleep [11]. In fact, a PAP clinical trial showed that treating OSA was associated with a 50% reduction in bedpartners’ nocturnal arousals [21]. Second, bedpartner’s sleep disruption is a primary motivator for patients to seek OSA diagnosis. In contrast, 50% of OSA patients reported they would not use PAP if it disrupted their partners’ sleep [22]. Third, evidence from other chronic illness populations (e.g., cancer, HIV, diabetes) shows that couples-based interventions [23] are effective at improving adherence, symptom management, and patient and partner health outcomes. Finally, partner support is critical to promote PAP adherence, whereas relationship conflict may reduce adherence [24, 25]. Our previous studies have shown that collaborative support (e.g., helping with the PAP machine) strongly predicted greater adherence, whereas pressure (e.g., nagging) to use CPAP [25] and relationship conflict predicted lower adherence [24].

Only one previous pilot investigation [26] examined use of a couples’ education and support intervention compared to patient-oriented education and usual care groups. Results demonstrated improvements in patient PAP adherence as well as moderate to large improvements in sleep quality and reductions in daytime sleepiness in patients and partners in the couples’ group; however, only 6 of 10 couples randomized to this condition completed the intervention, highlighting the challenge of having both couple members attend in-person sessions. Furthermore, this pilot study focused exclusively on enhancing PAP adherence as opposed to addressing sleep issues experienced by both members of the couple, which may have reduced partner motivation to participate. Collectively, these findings provide a strong theoretical rationale and preliminary empirical support for integrating the partner into PAP adherence interventions; however, new treatment approaches are needed to reduce couples’ burden and enhance treatment engagement.

Our proposed intervention is the first treatment for OSA that uses a couples-based treatment model to target PAP adherence, as well as the broader sleep health issues affecting both patient and partner. In doing so, we aim to address the limitations of prior studies by focusing on collaboration of the couple and addressing sleep health issues in both partner members, as opposed to exclusively focusing on the individual with OSA. Beyond OSA, older adults are at increased risk for poor sleep health, including insomnia/poor sleep quality, circadian disruptions (phase advances or irregular sleep/wake schedules), and irregular sleep-wake schedules, which can significantly increase risk for cardiometabolic disorders, cognitive decline, poor relationship functioning, and reduced QOL [27–31]. Further, OSA and insomnia commonly co-occur, with 22 to 55% having both conditions [32]. Moreover, partners of individuals who snore are three times more likely to have insomnia as compared to those living with non-snorers [21]. Finally, patients with insomnia have poorer PAP adherence [33], and partners’ sleep disturbances are associated with reduced PAP adherence [12]. Recent studies have tested insomnia treatments in patients with OSA and demonstrate promising results [32, 34, 35]. Importantly, no existing treatments focus on improving sleep health in partners of OSA patients, who are also a high-risk group for sleep disturbances [22]. Therefore, our intervention fills a critical gap by addressing sleep health in older adults with OSA and their partners, with the goal of improving PAP adherence, QOL, and the sleep and health of both partners.

Accordingly, the goal of this manuscript is the provide the theoretical framework, intervention details, assessments and analysis plan of the We-PAP intervention, a couples-based intervention aimed at improving PAP adherence and sleep health for both the patient and partner. This intervention aims to address barriers to couples-based treatment by focusing on collaboration for both partners’ sleep health and delivering a brief intervention via a convenient and user-friendly telehealth format.

## Design and methods

### Study design overview

**Fig. 1:**
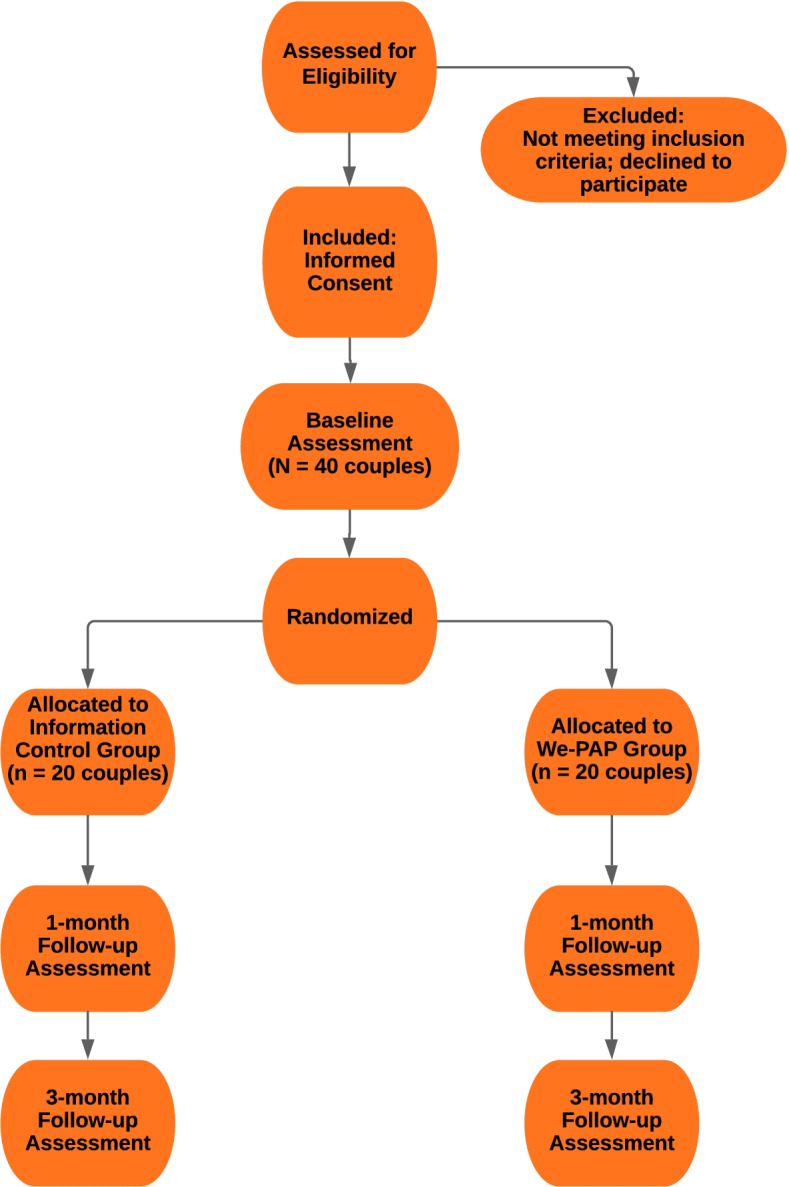
Flowchart of study design

The study flow diagram is listed in Fig. 1. This is a two-arm, parallel-group, single-blind, randomized controlled pilot trial to evaluate the feasibility and preliminary effectiveness of “We-PAP”, a novel, couples-based sleep health intervention for patients with OSA and their partners compared to an information control (IC). Participants include patients who are newly diagnosed with OSA and starting PAP therapy and their partners (*n* = 40 couples). After completing a pre-treatment baseline assessment, couples are randomly assigned to a 3-session online sleep health intervention (We-PAP) or to a patient-focused information control group (IC) arm. Patients and partners complete follow-up assessments at post-treatment (approximately 1 month after starting PAP) and 3 months after starting PAP treatment. Approval for this on-going study was provided by the University of Utah Institutional Review Board (IRB_000135927). The study is registered with ClinicalTrials.gov (NCT04759157, 2/13/2021).

### Theoretical framework

We-PAP is based on the transdiagnostic sleep and circadian model (i.e., a treatment that addresses common components of multiple disorders rather than a single disorder) [36]. This model uses the basic principles of sleep regulation (e.g., regulating sleep pressure and improving the regulation of the circadian system) as building blocks for improving sleep health for a variety of sleep conditions. In this study, we expanded this transdiagnostic framework to couples’ sleep health conceptualization, in that both partners’ sleep health as a “shared” experience. Our intervention contain three crosscutting themes, present in each session: education, dyadic coping and enhancing communication. Education includes education about sleep apnea, PAP treatment, and sleep health. Dyadic coping refers to the recognition that the illness (OSA) affects both members of the couple, which evokes a coping response in both members. Therefore, We-PAP helps OSA patients and partners conceptualize PAP adherence and both of their sleep health issues as a couple-level problem (i.e., a “we problem”). The enhancing communication theme focuses on promoting collaborative support within the couple and reducing conflict. Substantial research in couples shows that while a supportive partner is crucial for supporting an array of health behaviors, including diet, physical activity, sleep, and treatment adherence, a high conflict relationship is a primary source of stress, which can undermine health behaviors [11, 37–39].

### Recruitment, screening, and consent

A total of 40 couples will be recruited from the University of Utah Health Sleep Centers. Patients who are undergoing testing (in-lab or home sleep testing) are provided a letter introducing the study and notifying them they may be contacted about the study. Study staff contact potential patients via phone and email to provide study information and if interested, conduct screening for study eligibility. After completing screening, couples schedule an online consent visit over a HIPAA-compliant video conference platform (Zoom) to review and sign the online consent form and arrange to complete the baseline assessment. We enhanced our recruitment of patients from diverse backgrounds by using flyers with pictures of patients from different ethnicities and recruiting patients from suburban sleep clinics that serve a more diverse patient population.

### Eligibility criteria

Inclusion criteria for the couple include (1) age 50–85 years; (2) mixed or same sex couples who are married or living with a romantic partner for at least 1 year; (3) Patient is PAP naïve or re-initiating PAP after (3 or more years; (4) able to read and write in English; (5) Able to access online, video-conference capabilities (Wi-Fi or cellular plan)

Exclusion criteria include (1) self-reported diagnosis of severe comorbid sleep disorders other than insomnia (e.g., moderate or severe RLS; REM Behavior Disorder, narcolepsy); (2) presence of severe medical and psychiatric disorders that would interfere with participation in treatment (schizophrenia, bipolar disorder, hemodialysis); (3) patient is using supplemental oxygen or adaptive servo-ventilation.

### Inclusion criteria justifications

Our age criteria (age > 50) was selected because the focus of our project is on improving the treatment of OSA to promote healthy aging. We included both bed-sharing and non-bed sharing couples based on feedback from our preliminary focus groups. Provided reasons included that partners need more education and support in initiating and maintaining progress with PAP treatment regardless of whether they share a bed, as well as concerns about feasibility, given that many older adult couples with OSA choose to sleep apart. Patients who use supplemental oxygen or ASV are excluded due to the presence of additional comorbidities beyond OSA, such as cardiopulmonary disease or other hypoventilatory disorders that lead to more challenges in ensuring adequate optimization of PAP-based treatments.

### Randomization

Upon completion of the baseline assessment, couples are randomly assigned to either We-PAP or IC using the RedCAP randomization algorithm. The randomization table, developed by the statistician (BB), uses a 1:1 ratio of We-PAP to IC with equal strata for male and female patients using a random number generator. The study interventionist confirms with the study staff that the couple meet the eligibility criteria then contacts the couple via letter and email to notify them of their group assignment.

### Assessment schedule

At baseline/pretreatment, couples complete questionnaires, 7 days of wrist actigraphy and daily sleep diaries (delivered either via text message or on paper), and a brief cognitive testing battery. Approximately 1 month after beginning PAP and upon completion of their intervention, couples complete a second set of questionnaires. Finally, at 3 months after starting PAP, couples complete questionnaires, 7 days of actigraphy and daily sleep diaries and a brief cognitive testing battery. PAP use data are collected via download using the corresponding cloud-based system. The 3-month time point was selected as the primary endpoint because this is consistent with Medicare criteria for continuing coverage for PAP treatment. The 1-month assessment is included to evaluate the early changes with PAP because early adherence and improvement in outcomes are associated with longer term adherence [15].

### Intervention description

We-Pap was developed through an iterative process that included focus groups and a brief field trial (*n* = 4 couples) before beginning the RCT. This intervention is a novel, couples-focused PAP adherence and sleep health treatment that combines a transdiagnostic sleep and circadian framework with a dyadic (i.e., couples) perspective. Each couple assigned to the We-PAP intervention completes three online Zoom-hosted, sessions conducted weekly, utilizing structured PowerPoint slides. All sessions are structured, but interactive, and include the themes of sleep education, dyadic coping and communication. Each session has content geared at the patient, partner and the couple. A detailed list of session content is listed in Table 1.

- Session 1 (75 min) focuses on assessment and the couples’ sleep, knowledge about OSA, and expectations for beginning PAP. The main content of the session reviews education about PAP treatment including overcoming early challenges to PAP treatment and how to effectively communicate and elicit partner support for troubleshooting.
- Session 2 (60 min) focuses on sleep health and strategies to improve poor sleep (techniques based on Brief Behavioral Treatment for Insomnia; BBTI) [40] including improving sleep habits, reducing time in bed, stimulus control techniques, and setting a consistent rise time. Sleep diary data between Sessions 1 and 2 are reviewed.
- Session 3 (60 min) begins by reviewing the patient’s PAP download, discussing each person’s sleep diaries and adjusting the sleep window, then discusses the role of stress and hyperarousal, both in general and specific to adjusting to PAP, including techniques for PAP desensitization and establishing a wind-down routine for the couple that incorporates PAP as the final step of the routine.

**Table 1:** Session content

Session	Content
1	• **Program overview:** Impact of OSA on the patient and partner, what to expect in the sessions, treatment goals. • **Assessment:** Main symptoms/sleep challenges, current sleep routine, goals (individual and couple-focused) • **Education:** Background about sleep apnea and PAP, common problems with PAP, couple collaboration for troubleshooting PAP issues, communicating about PAP problems • **Homework:** Review goals, reminder to complete sleep diaries
2	• **Review homework:** Review goals, homework, sleep diaries, and PAP download; discuss and strategize any PAP problems • **Sleep health:** Defining sleep health and techniques for improving sleep health based on BBTI (consistent wake-time, stimulus control, sleep compression (as appropriate), sleep hygiene • **Homework:** Set action plan for sleep health improvement, complete sleep diaries
3	• **Review homework:** Review sleep diaries and PAP download, strategize any PAP problems. • **Sleep health:** Adjusting the sleep schedule (increase or decrease sleep opportunity window based on sleep efficiency) • **Evening routines:** Discuss the role of hyperarousal/stress in contributing to sleep disturbance, both in general and specific to PAP anxiety; delivery of relaxation techniques including: establishing a wind-down together and PAP desensitization

Cross-cutting themes (in each session): education, dyadic coping, and enhancing communication

*OSA* Obstructive Sleep Apnea, *PAP* Positive airway pressure, *BBTI* Brief behavioral treatment of insomnia

During each session, the interventionist reviews homework (if applicable), presents session content, engages the couple in discussion and planning and assigns homework for the next session (if applicable).

### Information control (IC)

In this group, couples receive a packet of standardized patient educational materials about OSA and PAP published by the American Academy of Sleep Medicine on the website Sleepeducation.org. The purpose of this group is to compare our novel intervention to a standardized usual care comparator. This group is considered an enhanced usual care group, because they are receiving usual care in the sleep clinic plus standardized medical information provided by the research study [41]. The interventionist will contact couples to notify them of their study assignment and then follow-up with them with a phone or email contact to ensure they have received the study materials. If the couple has questions about PAP, the interventionist refers couples to direct questions to their sleep medicine provider or durable medical equipment (DME) company.

### Interventionist training, supervision, and fidelity monitoring

All We-Pap intervention sessions are recorded for training and fidelity monitoring. The interventionist delivering the We-PAP intervention content has a doctoral degree in clinical psychology and is currently participating in a Behavioral Sleep Medicine Fellowship program. Training included didactics, supervised telehealth patient sessions focused on CBTi at the University of Utah Sleep Wake Center, rehearsal of We-Pap sessions prior to the field trial, role-playing, review of pre-recorded session format provided by the lead investigators, in-person review and coaching of digitally recorded field trial sessions, and weekly supervision meetings. The interventionist is supervised by one of the lead investigators (WT). Fidelity is monitored via a structured checklist completed by the therapist at the end of each session and 10% of sessions will be rated for fidelity by one of the lead investigators (WT or KB). The checklist also includes an open-ended comments section, where the interventionist writes notes to review in weekly meetings with the supervisor.

### Measures

Participants complete a brief demographic measure, including age, sex, race/ethnicity, marital status, education level, employment status, household income level, substance use, and bed-sharing.

OSA severity (AHI) will be extracted from medical records.

### Feasibility and acceptability measures

Our primary outcomes assess feasibility of enrollment and retention including (1) our ability to meet recruitment goals (4 couples per month) and (2) percentage of participants completing all three intervention sessions. We also collect other quantitative ratings of feasibility and acceptability of the treatment including Likert scale ratings of ease of participation in the telehealth, and perceived benefits of the intervention and enjoyment of the sessions. Additionally, we collect qualitative data via open-ended questions about the format, feedback on the content, materials and general feedback and suggestions for improving the interventions. Success will be defined as meeting recruitment goals of our study (enrolling 100% of the planned sample) and retention of 85% of our participants in the intervention.

### Secondary outcome measures

PAP adherence (main secondary outcome)–patient PAP adherence is recorded by the patient’s PAP machine and remotely downloaded. PAP adherence is measured continuously. The main time point for adherence is at 3 months. The average duration of use per night, percent of nights with use > 4 h, and nights skipped is recorded.

### Objective sleep measures

Sleep is estimated using the Actiwatch Spectrum Plus (Philips Respironics, Murrysville, PA, USA). Actiwatches are configured with default settings using 30-s epochs. Rest intervals are manually set with assistance of a sleep diary (to indicate bedtimes and wake times). Using the Actiware software, we calculate total sleep time, sleep onset time, sleep offset time, sleep duration, sleep efficiency, sleep latency, wake after sleep onset, and sleep fragmentation index. The main measure of sleep quality is sleep efficiency.

### Sleep diary

Participants complete the Consensus Sleep Diary [42] to quantitatively assess dimensions of sleep that are important for a wide-range of clinical and research applications. Items include time the individual got into and out of bed, time the individual tried to go to sleep, duration to fall asleep, and frequency and total duration of awakenings, time of final awakening and sleep quality rating. Two additional items included in the diary assess whether partners shared a bed (yes/no) and the degree to which the couple worked together to use PAP (rated on a scale 1–5).

### Sleep disturbance and sleep-related impairment

Participants complete the PROMIS sleep disturbance and sleep-related impairment adaptive measures [43] Scores are presented as *t* scores, with average of 50 and SD of 10. Scores > 60 are considered elevated. Self-reported sleep is measured using a standardized sleep diary and the PROMIS sleep disturbance questionnaire.

### Other outcome measures

#### Relationship quality

Participants complete the Couples Satisfaction Index (CSI-4) [44] to evaluate an individual’s self-reported degree of satisfaction, happiness, warmth and comfort, and reward with the relationship. The 4-items on the scale are summed and range from 0 to 21. Higher scores indicate higher levels of relationship satisfaction. CSI-4 scores falling below 13.5 suggest notable relationship dissatisfaction.

### Cognitive function

A study staff member administers the Repeatable Battery for the Assessment of Neuropsychological Status (RBANS) forms A and B via videoconference (Zoom) [45]. This 30 min validated clinical measure was designed to assess cognitive status in adults and older adults. The five sub-scores include attention, language, visuospatial/construction, immediate and delayed memory as well as a total score. In order to complete this measure via teleconference, couples are mailed the coding subtest in a sealed envelope and told not to open it until the assessment. Then, they are asked to place the completed test in a sealed envelope during the testing session.

### Statistical analysis

Statistical analyses will be conducted using Stata v15 or higher. We will begin with conducting descriptive analyses, including evaluating whether there are any baseline group differences. Any variables that emerge as being significantly different between the two treatment groups will be included as covariates in models predicting outcome variables. If more than three variables emerge as being significantly different between the treatment arms, the full set of demographics and sleep related functioning variables are used to estimate a propensity score indicating likelihood of membership in We-PAP relative to information control (IC); this propensity score is included as a covariate in all tests of study outcomes. Given the pilot nature of the proposed study, significance tests of study hypotheses are performed using one-sided tests with α = 0.10. Feasibility analysis will include review of the adherence and treatment satisfaction measures. Models testing preliminary efficacy will be conducted using multilevel models with separate models will be run for each outcome. The primary outcome is PAP adherence. Secondary outcomes are objective and self-reported sleep quality. Tertiary outcome measures will be explored including relationship satisfaction and cognitive outcomes. Missing data: Mechanisms of missingness will be evaluated in outcomes and potential mediators with missing data. If missingness patterns are consistent with MAR assumptions, recently developed fully conditional specification with chained equations methods for multiple imputation of missing data in MLMs will be used to handle missing data [46, 47]. All variables in hypothesis tests plus additional auxiliary variables will be included; potential auxiliary variables will be carefully screened using methods recommended for small sample sizes [48]. If missingness patterns are consistent with MNAR assumptions, we will consider the use of pattern mixture models [49] and conditionally dependent dropout [50] models to minimize any potential bias in estimation of treatment effects.

### Sample size

We selected a sample size of 40 couples (20 per arm) based on power analyses of secondary outcomes with additional consideration of required group sizes for stable and precise estimates of treatment effects and feasibility constraints of the funding mechanism. Although there is some debate about sample size needed to have adequate power for detecting feasibility outcomes [51], we selected a sample size that will allow us to (1) evaluate the feasibility of enrollment and retention needed to conduct a successful fully powered clinical trial and (2) evaluate preliminary efficacy of our main sleep outcome of interest (PAP use). Power estimates were generated using descriptive statistics for behavioral and cognitive behavioral CPAP interventions vs. IC reported in Wozniak et al. [52]. Power estimates based on these assumptions indicate that a sample size of 20 couples per group provides power of .8 or higher to detect between group differences of *d* = .68 for average hours of CPAP usage and O.R. = 4.36. Power estimates for patient and partners’ sleep [actigraphy and diary-assessed] were generated using descriptive statistics for behavioral and cognitive behavioral CPAP interventions vs. IC as reported in Wozniak et al. [52] as well as in Buysse et al. [53] and Sweetman et al. [54] Maas and Hox’s (2005) [55] canonical work on necessary sample sizes for group level effects in MLM determines that group sizes of 20 members and larger is sufficient for generating unbiased estimates for our secondary measures (Table 2).

**Table 2:** Study schedule of enrollment, allocation, interventions, and assessment

	**Study period**					
**Timepoint**	Enrollment	Pre-intervention assessment	Allocation	**Post-allocation**		
				Intervention	Post-intervention 1-month assessment follow-up	Post-intervention 3-month assessment follow-up
**Enrollment**						
Eligibility screen	X					
Informed consent	X	X				
Allocation			X			
**Intervention**						
We-PAP group				X		
Information control group				X		
**Data collection**						
Demographics		X				
OSA severity (AHI)		X		X		X
**Primary outcomes**						
PAP adherence		X		X		X
**Secondary outcomes**						
Sleep quality (objective)		X		X		X
Sleep diary		X		X		X
Sleep disturbance		X		X	X	X
Sleep-related impairment		X		X	X	X
Relationship quality		X		X	X	X
Cognitive function		X				X
**Process/intervention-related variables**						
Feasibility/acceptability				X	X	X
Intervention fidelity				X		

## Discussion

The We-PAP study is the first intervention designed to improve PAP adherence and overall sleep health for older adult patients with OSA and their partners. This intervention was developed in collaboration with patients, partners, and sleep medicine providers and aims to create a collaborative approach to PAP adherence and sleep health for both the patient and partner. The brief 3-session format and the delivery of the intervention via telehealth is designed to maximize accessibility, by allowing couples to participate from their home at a convenient time.

The significance of this intervention is that We-PAP represents a new conceptual approach to treatment of patients of OSA, by taking a couples-based and transdiagnostic approach to improving sleep health for both the patient and partner. This pilot trial will provide important information regarding the feasibility and acceptability of this intervention. We will recruit equal numbers of male and female patients as well as participants from diverse backgrounds. Even in the first few months, we have been able to enroll couples from rural areas located three or more hours from the clinic, which is important because these patients have greater barriers to attending follow-up visits. We will also be powered to evaluate preliminary efficacy for PAP adherence and improvement in sleep quality.

Limitations to this study include lack of a robust plan for recruiting diverse participants. In a larger, fully powered study, we will plan to recruit from multiple sites, thus increasing the race/ethnic and geographic diversity of our sample. We will also administer our intervention in Spanish, which will extend our intervention to Utah’s largest minority population (> 20% of the population of Salt Lake City). In addition, in larger studies we will have the ability to determine mechanisms of the treatment effect, such as whether changes in relationships or dyadic coping predict changes in adherence.

If successful, our next steps will be to test the We-PAP intervention in a fully powered RCT to evaluate the We-PAP intervention to treatment as usual in a larger and more diverse patient sample. This intervention has the potential to improve aging-related health and cognitive outcomes for older adults. This intervention may also be useful for patients with cognitive difficulties, including mild cognitive impairment (MCI) and Alzheimer’s disease (AD), given that PAP adherence in these populations has the potential to improve cognitive function.

## Data Availability

N/A

## Abbreviations

OSA: Obstructive Sleep Apnea
PAP: Positive Airway Pressure
IC: Information control
